# Supplementary material for: Characterizations of novel pesticide-degrading bacterial strains from industrial wastes found in the industrial cities of Pakistan and their biodegradation potential
Source: PeerJ. 2021 Oct 5;9:e12211. doi: 10.7717/peerj.12211 (PMC8500106; doi:10.7717/peerj.12211)
Supplement: Supplemental Information 1 [file peerj-09-12211-s001.docx]

**Supplementary data**

**
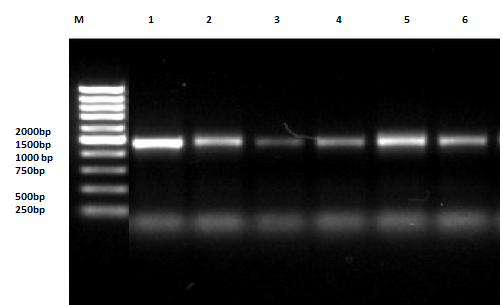
**

**Figure S1. PCR amplification of 16S rRNA of different isolates Lanes: M: 1Kb- DNA marker; 1: 1A; 2: 2B; 3: 3C; 4: 4D; 5: 5E, 6: 6F**

**Table S1 -Biodegradation of DDT**

| **Strains** | **Time (hrs)** | | | | | | | | | **p value** |
| --- | --- | --- | --- | --- | --- | --- | --- | --- | --- | --- |
|  | 2 | 6 | 8 | 12 | 16 | 20 | 24 | 30 | 36 |  |
| **1A** | 0.00 | 1.733333 | 15.333333 | 23.5 | 34.0 | 39.0 | 53.0 | 69.0 | 76.0 | < .00001 |
| **2B** | 0.00 | 1.833333 | 13.333333 | 25.66667 | 33.83333 | 41.0 | 53.0 | 70.66667 | 70.0 | < .00001 |
| **3C** | 0.00 | 1.7 | 15.0 | 26.66667 | 34.33333 | 45.0 | 56.0 | 70.83333 | 71.66667 | < .00001 |
| **4D** | 0.00 | 2.433333 | 7.6666667 | 24.66667 | 33.66667 | 47.33333 | 55.0 | 68.0 | 75.66667 | < .00001 |
| **5E** | 0.00 | 1.066667 | 4.3333333 | 11.16667 | 15.0 | 24.0 | 30.33333 | 34.33333 | 37.33333 | < .00001 |
| **6F** | 0.00 | 1.133333 | 2.5 | 5.666667 | 9.666667 | 15.33333 | 17.33333 | 21.33333 | 27.66667 | < .00001 |

**Table S2 - Biodegradation of Alderin**

| Strains | Time (hrs) | | | | | | | | | p value |
| --- | --- | --- | --- | --- | --- | --- | --- | --- | --- | --- |
|  | 2 | 6 | 8 | 12 | 16 | 20 | 24 | 30 | 36 |  |
| **1A** | 0.00 | 0.00 | 7.666667 | 14.33333 | 24.33333 | 31.66667 | 42.33333 | 49.66667 | 57.33333 | < .00001 |
| **2B** | 0.00 | 2.0 | 10.0 | 21.66667 | 32.66667 | 44.0 | 59.0 | 65.33333 | 68.33333 | < .00001 |
| **3C** | 0.00 | 2.0 | 15.33333 | 28.33333 | 36.33333 | 45.33333 | 57.33333 | 73.0 | 78.0 | < .00001 |
| **4D** | 0.00 | 3.666667 | 8.333333 | 23.66667 | 32.33333 | 45.66667 | 56.0 | 65.33333 | 71.66667 | < .00001 |
| **5E** | 0.00 | 0.00 | 4.333333 | 6.333333 | 10.33333 | 20.33333 | 24.0 | 30.33333 | 40.0 | < .00001 |
| **6F** | 0.00 | 0.00 | 2.333333 | 5.666667 | 12.33333 | 16.33333 | 20.33333 | 24.66667 | 30.33333 | < .00001 |

**Table S3- Biodegradation of Malathion**

| **Strains** | **Time (hrs)** | | | | | | | | | **p value** |
| --- | --- | --- | --- | --- | --- | --- | --- | --- | --- | --- |
|  | 2 | 6 | 8 | 12 | 16 | 20 | 24 | 30 | 36 |  |
| **1A** | 0.00 | 2.0 | 10.0 | 17.33333 | 24.0 | 26.0 | 28.33333 | 30.33333 | 32.66667 | 0.000213 |
| **2B** | 0.00 | 2.0 | 12.0 | 19.33333 | 26.0 | 28.0 | 32.66667 | 34.33333 | 35.66667 | 0.000226 |
| **3C** | 0.00 | 3.0 | 12.0 | 21.0 | 25.0 | 30.0 | 32.66667 | 35.66667 | 40.66667 | 0.000049 |
| **4D** | 0.00 | 2.0 | 8.6666667 | 17.66667 | 26.0 | 30.0 | 31.0 | 34.66667 | 36.66667 | .00013. |
| **5E** | 0.00 | 4.0 | 8.3333333 | 19.33333 | 27.666667 | 40.0 | 48.0 | 50.66667 | 52.66667 | 0.000019 |
| **6F** | 0.00 | 4.0 | 10.666667 | 23.33333 | 31.333333 | 42.0 | 48.66667 | 52.33333 | 54.33333 | 0.000026 |

**Table S4-Growth rate of Biodegrading bacteria in MSM broth at different temperatures**

| **At 15°C** | | | | | | | | | |
| --- | --- | --- | --- | --- | --- | --- | --- | --- | --- |
| **Strains** | **TIME (hrs)** | | | | | | | | **P value** |
|  | **2** | **6** | **8** | **16** | **20** | **24** | **30** | **36** |  |
| **1A** | 0.00123333 | 0.07666667 | 0.22666667 | 0.57 | 0.64333333 | 0.78 | 0.96333333 | 2.13666667 | .00106 |
| **2B** | 0.0011 | 0.06666667 | 0.22333333 | 0.45666667 | 0.54333333 | 0.63 | 0.89 | 2.2 | 0.003232 |
| **3C** | 0.00133333 | 0.05333333 | 0.19333333 | 0.53666667 | 0.62666667 | 0.77 | 0.96333333 | 2.16666667 | 0.001161 |
| **4D** | 0.00133333 | 0.06666667 | 0.21333333 | 0.61333333 | 0.81333333 | 0.87333333 | 1.02333333 | 2.16666667 | 0.000493 |
| **5E** | 0.00166667 | 0.07333333 | 0.22333333 | 0.62666667 | 0.71666667 | 0.75333333 | 0.78333333 | 2.13333333 | 0.002627 |
| **6F** | 0.001 | 0.07 | 0.22333333 | 0.43 | 0.71666667 | 1.16333333 | 1.4 | 2.26666667 | 0.000095 |
| **At 30°C** | | | | | | | | | |
| **1A** | 0.41333333 | 0.43666667 | 0.51333333 | 0.3 | 0.63 | 0.80333333 | 1.33 | 1.14666667 | 0.007416 |
| **2B** | 0.23 | 0.33666667 | 0.40333333 | 0.41333333 | 0.72 | 0.89666667 | 1.38 | 1.19333333 | 0.000508 |
| **3C** | 0.39333333 | 0.45333333 | 0.52333333 | 0.51 | 0.75666667 | 0.75333333 | 1.53666667 | 1.13 | 0.006858 |
| **4D** | 0.35 | 0.44 | 0.60666667 | 0.80333333 | 0.83333333 | 1.2 | 1.43 | 1.21 | 0.000455 |
| **5E** | 0.40666667 | 0.32333333 | 0.52333333 | 0.72666667 | 0.73333333 | 1.16666667 | 1.64333333 | 0.85333333 | 0.025692 |
| **6F** | 0.4 | 0.21 | 0.31333333 | 0.33333333 | 0.69666667 | 1.13333333 | 1.34666667 | 1.88666667 | 0.000943 |
| **At 45°C** | | | | | | | | | |
| **1A** | 0.05333333 | 0.59 | 0.74 | 0.81333333 | 1.13 | 1.33666667 | 1.84 | 2.62333333 | 0.000112 |
| **2B** | 0.05 | 0.57666667 | 0.69666667 | 1.21666667 | 1.25666667 | 1.43666667 | 2.13333333 | 2.78666667 | 0.000017 |
| **3C** | 0.06666667 | 0.56666667 | 0.74 | 0.92333333 | 1.34333333 | 1.32333333 | 2.16666667 | 2.62666667 | 0.000035 |
| **4D** | 0.04 | 0.55 | 0.74333333 | 1.31666667 | 1.34333333 | 1.60333333 | 1.91333333 | 2.50666667 | < .00001 |
| **5E** | 0.05666667 | 0.58 | 0.73666667 | 1.22666667 | 1.22666667 | 1.71666667 | 2.13666667 | 2.71333333 | < .00001 |
| **6F** | 0.04666667 | 0.56666667 | 0.77 | 0.84666667 | 1.21333333 | 1.71 | 1.8 | 2.69 | 0.000064 |
